# Acknowledgement to Reviewers of *Antibiotics* in 2014

**DOI:** 10.3390/antibiotics4010042

**Published:** 2015-01-08

**Authors:** Antibiotics Editorial Office

**Affiliations:** MDPI AG, Klybeckstrasse 64, CH-4057 Basel, Switzerland

The editors of *Antibiotics* would like to express their sincere gratitude to the following reviewers for assessing manuscripts in 2014:

Aabenhus, Rune
Aldeyab, Mamoon
Ann, Progulske-Fox
Arthur, Michel
Ashour, Hossam
Ayyalusamy, Ramamoorthy
Van Bambeke, Françoise
Bergman, Peter
Bevins, Charles L.
Bonomo, Robert A.
Bordon, Jose M.
Brackman, Gilles
Butler, Mark S.
Chang, Theresa Li-yun
Chen, Feng-Jui
Chris, Del Mar,
Cody, Vivian
Cole, Alexander
Cotta, Menino O.
Diehnelt, Chris W.
Drlica, Karl
Dupont, Bertrand
Ernst, Robert
Fadl, Amin
Fisher, Jed F.
Gebhard, Susanne
Geisler, William M.
Giacomini, Daria
Goldberg, Joanna B.
Gonzalez, Ralph
Grossman, Trudy H.
Hughes, Diarmaid
Jacobs, Bill
Jelokhani-Niaraki, Masoud
Jimi, Shiro
Johansen, Truls
Johnson, Georgia
Jordana, King
Kaase, Martin
Kaur, Kamaljit
Kielian, Tammy
Koller, D.
Komatsuzawa, Hitoshi
Krueger, Christian G.
Kurwadkar, Sudarshan
Kyselkova, Martina
Livesay, Dennis R.
Madhyastha, Srinivasa
Marques, Claudia
Mason, Kevin
Matsumoto, Tetsuro
McBride, Shonna M.
Mclean, Robert J.C.
Minardi, Daniele
Montelaro, Ronald
Mookherjee, Neeloffer
Naber, Kurt
Nagant, Carole
Nicolle, Lindsay E.
Nolan, Elizabeth M.
Odagiri, Takashi
Oelschlaeger, Peter
Ouellette, Andre
Ouellette, Andrew
Pini, Alessandro
Powers, Rachel A.
Rada, Balazs
Salzman, Nita
Sass, Peter
Selan, Laura
Shafer, William M.
Shai, Yechiel
Shigemura, Katsumi
Simerska, Pavla
Simpson, Jamie
Sougakoff, W.
Stegmann, Evi
Sun, Peizhe
Thaker, Maulik N.
Thomas, Hermann
Tokuriki, Nobuhiko
Tossi, Alessandro
Van Der Velden, Alike
Van Dijck, Patrick
Van Hoek, Monique L.
Viveiros, Miguel
Vogel, Hans
Weiss, David
White, Steve
Zander, Esther
Zarb, Peter
Zhang, Ying

